# Optimal Surface Amino-Functionalization Following Thermo-Alkaline Treatment of Nanostructured Silica Adsorbents for Enhanced CO_2_ Adsorption

**DOI:** 10.3390/ma9110898

**Published:** 2016-11-04

**Authors:** Obdulia Medina-Juárez, Miguel Ángel García-Sánchez, Ulises Arellano-Sánchez, Isaac Kornhauser-Straus, Fernando Rojas-González

**Affiliations:** Department of Chemistry, Universidad Autónoma Metropolitana-Iztapalapa, Av. San Rafael Atlixco 186, Col. Vicentina, México City 09340, Mexico; mjyuyu@gmail.com (O.M.-J.); mags@xanum.uam.mx (M.Á.G.-S.); lukas261100@hotmail.com (U.A.-S.); iks@xanum.uam.mx (I.K.-S.)

**Keywords:** CO_2_ enhanced capture, SBA-15 habilitation for CO_2_ sorption, desilication, silanol functionalization, covalent coordinated CO_2_ deposition

## Abstract

Special preparation of Santa Barbara Amorphous (SBA)-15, mesoporous silica with highly hexagonal ordered, these materials have been carried out for creating adsorbents exhibiting an enhanced and partially selective adsorption toward CO_2_. This creation starts from an adequate conditioning of the silica surface, via a thermo-alkaline treatment to increase the population of silanol species on it. CO_2_ adsorption is only reasonably achieved when the SiO_2_ surface becomes aminated after put in contact with a solution of an amino alkoxide compound in the right solvent. Unfunctionalized and amine-functionalized substrates were characterized through X-ray diffraction, N_2_ sorption, Raman spectroscopy, electron microscopy, ^29^Si solid-state Nuclear Magnetic Resonance (NMR), and NH_3_ thermal programmed desorption. These analyses proved that the thermo-alkaline procedure desilicates the substrate and eliminates the micropores (without affecting the SBA-15 capillaries), present in the original solid. NMR analysis confirms that the hydroxylated solid anchors more amino functionalizing molecules than the unhydroxylated material. The SBA-15 sample subjected to hydroxylation and amino-functionalization displays a high enthalpy of interaction, a reason why this solid is suitable for a strong deposition of CO_2_ but with the possibility of observing a low-pressure hysteresis phenomenon. Contrastingly, CH_4_ adsorption on amino-functionalized, hydroxylated SBA-15 substrates becomes almost five times lower than the CO_2_ one, thus giving proof of their selectivity toward CO_2_. Although the amount of retained CO_2_ is not yet similar to or higher than those determined in other investigations, the methodology herein described is still susceptible to optimization.

## 1. Introduction

### 1.1. The Problem

The effective and abundant capture of CO_2_ on the surface of nanoparticulate substrates is a fundamental experimental aim, since this gas plays a leading role in the upsurge of the greenhouse effect; this last phenomenon being nowadays one of the most important ecological problems to deal with. Several strategies for achieving satisfactory CO_2_ levels of capture already exist; one implementation of particular and novel interest consists in the trapping and eventual in situ transformation of this gas into useful and environmentally friendly compounds, via chemical procedures. Hence, with the objective in mind of reaching an efficient carbon capture and storage (CCS) [1,2,3], three alternatives are available at present: (a) CCS pre-combustion of carbon sources before actual fuel combustion; (b) oxycombustion (i.e., carbon compound combustion under nearly pure oxygen); and (c) post-combustion capture. Carbon capture after combustion is unavoidable due to the amount of industrial processes that depend on the overwhelming burning of fossil fuels; an example of this being cement production among many other processes involving the burning of huge amounts of carbon. Nowadays, the vast majority of CCS research is related to the occurrence of the following phenomena: (a) absorption [4,5]; (b) cryogenic separation [6]; (c) separation through membranes [7]; and (d) adsorption [8,9,10]. The materials employed to perform after combustion CCS operations require, besides being selective, some other crucial characteristics such as: (i) to be endowed with a high compound capture capacity; (ii) to be renewable; (iii) low cost; (iv) durable; and (v) practical.

### 1.2. Carbon Capture and Storage (CCS) Alternatives

In particular, several sorption experiments are very useful as CCS alternatives. According to the nature of the substrate employed, CO_2_ sorption or capture occurs through two possible processes:

- Absorption processes, which involve the bubbling of the adsorptive in solutions containing species such as amines, carbonates, and ions. In this case, the solvent becomes very important, since ion order to achieve a good capture of CO_2_, solvents of a low polarity and relatively low vapor pressure are very much preferred. Nevertheless, absorption processes involve a great disadvantage that is concerned with the short lifetimes that the trapped species can attain as these molecules usually interact (react) with the solvent species.
- Adsorption processes implying the employment of structures such as zeolites [11], hydrotalcites [12], polymer fibers [13], activated carbons [14], metal oxide networks [15], functionalized nanoporous materials [16,17,18], and, recently, metal oxide frameworks (MOFs) [19,20,21,22].

### 1.3. CO_2_ Chemisorption and Physisorption

The substrates that are more usually chosen for CO_2_ chemisorption capture are metal oxides (e.g., CaO and MgO); these solids can form carbonates in the presence of CO_2_. However, a shortcoming arises with the employment of these substrates, in view of the fact that high temperatures are required for achieving their total regeneration, i.e., 287 and 800 °C for MgO and CaO, respectively [15]. Lithium salts, such as Li_2_ZrO_3_ and Li_4_SiO_4_ [23,24], have been used to capture CO_2_ (between 450 and 700 °C), thus apparently being good alternatives for reaching an efficient CO_2_ adsorption, irrespectively of the high production cost involved with this kind of substrates.

Another fine alternative for effective CO_2_ chemisorption is the use of hydrotalcites, ever since León et al. [25] demonstrated that the irreversible reaction between these minerals and CO_2_ is due to the creation of unidentate adsorption sites. Likewise, the formation of bidentate sites generates surface bicarbonates, which behave as weak adsorption sites, useful for highly reversible reactions. The concentration of the adsorbate phase is closely related to the number of irreversible adsorption sites.

Active carbon possesses attractive characteristics for the physisorption of diverse species such as high uptake capacity, hydrophobicity and low cost; however, this substrate shows a poor selectivity between CO_2_ and N_2_. Lu et al. [26] observed that carbon nanotubes depicted a higher CO_2_ adsorption than zeolites when surface functionalized with amine groups (NH_2_) through the attachment of 3-aminopropyltriethoxysilane (APTES). Due to the previous arguments, a convenient adsorbent for CO_2_ capture depends on the temperature and pressure at which the adsorption process is to take place. According to these requirements, surface-functionalized mesoporous materials stand apart from the rest of adsorbents because of their ability to physisorb or chemisorb diverse compounds, depending on their physicochemical interactions (weak or strong) with the adsorbent.

Recently, the implementation of MOFs has brought about great advantages: (i) a good structural stability; and (ii) a high porosity this characteristic being reflected in surface areas of about 2900 m^2^·g^−1^ and large total pore volumes between 1 and 2 cm^3^·g^−1^ [19] if compared to solids such as zeolites and molecular sieves. However, a great disadvantage surges when trying to employ MOFS for CO_2_ adsorption, in view of the low selectivity of these solids towards CO_2_, especially when this adsorptive is mixed with other gases. Additionally, these materials are overwhelmed by high synthesis costs; furthermore, the capture capacity of MOF’s decreases under humid conditions, even though this last event could be dealt with through surface functionalization via diverse organic groups [20,21,22].

## 2. Stages Followed Concerning the Choice and Implementation of Proficient Silica Adsorbents for Enhanced CO_2_ Capture

### 2.1. Adsorbent Choice

The CO_2_ molecule is a Lewis acid since this molecule can accept a pair of electrons from a base in order to establish a covalent coordinated bond. Therefore, the crucial steps for designing a proper CO_2_ adsorbent include the selection of a substrate whose surface can be habilitated to possess a good number of basic sites; i.e., the surface has to be endowed with chemical species that can be combined with other matching molecules for attaining the desired characteristics.

Nanoporous silica supports [16,17,18] are provided, in general, with a high surface area and a considerable total pore volume. These characteristics make these substrates appropriate for adsorbing an assortment of chemical species. The employment of amphiphilic triblock copolymers as pore templating agents has allowed the creation of well-ordered mesoporous networks, which have been labeled as SBA-n (Santa Barbara) materials [27]. Among them, the SBA-15 [28] substrate results to have an ideal surface for performing an efficient and enhanced CO_2_ adsorption. On the one hand, this material is formed by a collection of cylindrical capillaries, all practically having the same diameters, in a hexagonal arrangement and lying parallel to each other along micrometric lengths. On the other hand, the capillaries open at both ends that are forming these mesoporous structures are endowed of such sizes as to allow the free transit of different molecules along the nanocylinder axis. This is a very important property since the adsorptive molecules can enter, while the adsorbate molecules can exit the structure easily.

Besides, the functionalization of the surface via, for instance, amino-alkoxides (i.e., the type of compounds that we will be employing for the amination of the SiO_2_ surface) can also benefit from this size characteristic, given that the deposition of molecules usually occurs after an adsorption from solution process. Another important property of amorphous silica is the presence of silanol (Si–OH) species throughout the surface. These species are determinant when the surface is to be functionalized with a given molecule. In the case of CO_2_, this molecule is in itself a weak Lewis acid, while the Si–OH groups are likewise. Then, in order for the silica surface to capture CO_2_, it has to be functionalized with pertinent species, e.g., amino-substituted alkoxides. The alkoxide groups can be attached to the surface after reacting with the silanol species; in turn, the CO_2_ molecules become coordinated to the NH_2_ groups of the amino-functionalizing compound.

### 2.2. Habilitation of the SiO_2_ Surface to Attain an Efficient and Sufficient Amine-Functionalization Process

Silica by itself manifests no significant affinity toward CO_2_ adsorption, for this reason, it is necessary to perform the chemical modification of this surface. This transformation can rely on the silanol groups, since the character and concentration of these species are crucial for attaining a widespread amino-functionalization of the SiO_2_ surface. Importantly, because of the thermal treatment required to eliminate remnant pore templating species, a relatively small population of silanol groups (–SiOH) subsists in the final network. Therefore, a hydroxylation process via treatment of the SiO_2_ substrate with a lowly concentrated NaOH solution, set at the right temperature, has been found to be a suitable operation for achieving an efficient hydroxylation of the surface. The reason of the low NaOH concentration to be used in this process resides in preventing an excessive desilication (i.e., dissolution and leaching) of the SiO_2_ matrix when put in contact with an alkaline solution [29]. Nonetheless, the previous chemical procedure can still result useful, not only for attaining more SiOH species on the surface, but to eliminate the micropores present in the parent silica material.

### 2.3. Supplementary Aspects in Relation to the Choice of Santa Barbara Amorphous Silica No. 15 (SBA-15) for Enhanced CO_2_ Trapping

Other aspects related to the convenience of choosing SiO_2_ as substrate for CO_2_ adsorption are the possibility of readily regenerating the adsorbent surface by means of a mild thermal treatment in an oven, thus reducing the power requirements necessary to perform the surface regeneration process. Another striking characteristic is the selectivity that the silica surface shows toward the adsorption of CO_2_ instead of adsorptives such as CH_4_.

Furthermore, the deposition of a functionalizing molecule on the silica surface can be made from either an organic or inorganic solution of the species in question. In the case of amine-functionalization, it is better to perform this process employing an appropriate organic liquid for dissolving the amino alkoxide compound chosen for functionalizing the surface, e.g., toluene instead of ethanol. The reason of this is the chemical character of the silanol groups, which can interact synergistically or not with the solvent molecules with respect to an effective deposition of the functionalizing compound [30].

The objective of this work consists in showing evidence of a more abundant and efficient CO_2_ capture on the basis of some appropriate textural changes that can be inflicted on model SBA-15 silica adsorbents via thermal and chemical surface functionalization procedures. A concomitant aim is to increase the number of silanol groups (i.e., –Si–OH) on the silica surface, since this is a key aspect for achieving an efficient functionalization of the surface via chemical anchorage methods. The ultimate goal is to accomplish an abundant capture of miscellaneous adsorptives (other than CO_2_) for their storage and eventual transformation into other molecules of diverse practical interest.

This work is organized as follows. First, the experimental section detailing the synthesis of the silica substrates is presented. Next, the thermo-alkaline habilitation of the surface of these solids is described. The following step consists in carrying out the functionalization of the previous materials with an especially selected amino-alkoxide compound. Subsequently, N_2_ sorption isotherms at 76 K are shown together with a list of the most important structural parameters of the porous solids involved in this investigation. CO_2_ adsorption isotherms, the center of this research, in the range of 263–313 K on the diverse SBA-15 materials are presented. Additionally, Fourier Transform Infrared (FTIR), X-ray Diffraction (XRD), Transmission Electron Microscopy (TEM), Thermogravimetric Analysis (TGA), solid-state Nuclear Magnetic Resonance (NMR), and Temperature Programed Desorption (TPD) characterization experiments are depicted. Finally, CH_4_ adsorption isotherms at 298 K on some of the SBA-15 adsorbents are presented, in order to ascertain the selectivity of these substrates between this last adsorptive and CO_2_.

## 3. Materials and Methods

### 3.1. Materials

Tetraethyl orthosilicate, TEOS (Aldrich, Saint Louis, MO, USA, 98%), hydrochloric acid (J.T. Baker, Chemicals, Center Valley, PA, USA, 36.5%–38.0% *w*/*w*), poly(ethylene glycol) Aldrich (for molecular biology), extra pure sodium hydroxide pellets (Riedel-de Haën, Lower Saxony, Germany, 99%–100%), 3-Aminopropyltriethoxysilane (APTES, of Sigma-Aldrich, 99%), toluene (Merck, Kenilworth, NJ, USA) and denatured ethyl alcohol 95% *v*/*v*, Pluronic 123 (BASF, Florham Park, NJ, USA) were used.

### 3.2. Synthesis of Precursor SBA-15 Solids

The following molar ratio sequence was employed to prepare SBA-15 silica:TEOS:197H_2_O:6.2HCl:0.017P123, the synthesis was performed according to the method devised by Beck et al. [24] and Zhao et al. [31]. The ensuing material was washed, filtered, put in contact with ethyl alcohol under stirring, and dried for 14 h at 373 K. Finally, the solid was calcined at 723 K for 6 h, while employing a heating ramp of 1 K·min^−1^.

### 3.3. Soft Thermo-Alkaline Treatment Performed over SBA-15 Precursor Materials

The mesoporous silica adsorbents of the SBA-15 (S15 denoted) kind were subjected to a thermo- (313 K) alkaline treatment through the employment of diluted (<1 M) NaOH solutions. Specifically, a 250 mg sample of each S15 substrate was subjected to a series of treatments under the previous conditions, each of these operations taking place every two hours. Subsequently, a desilication (i.e., hydroxylation) process was performed by adding a 0.025 M NaOH solution at 313 K (2 h). The resultant solid was washed with deionized water, filtered, and dried at 373 K for 2 h. The ensuing substrate was labeled as S15H.

### 3.4. Chemical Anchoring of 3-Aminopropyltriethoxysilane (APTES) on the Surface of SiO_2_ Mesoporous Solids

The chemical bonding of APTES on the surfaces of the S15 and S15H silica substrates was achieved once these solids were calcined in air at 723 K for 2 h. The advantage of this route is to guarantee the total removal of the surfactant molecules after the substrate has been annealed at 723 K.

Before APTES surface deposition, the precursor SiO_2_ solids were treated at 473 K under vacuum for 2 h in order to remove organic impurities and also to promote the activation of the silanol (SiOH) groups already residing on the surface. The molar ratio involved in this functionalization reaction corresponded to 3SiO_2_:2APTES and this procedure was carried out at 353 K under a N_2_ atmosphere and continuous magnetic stirring of a reflux system during 20 h, while employing toluene as the APTES dispersion medium. The final solids were separated by filtration and dried at 373 K for 2 h. These materials were labeled as S15NH_2_ and S15HNH_2_, given that these aminated materials proceed from the respective S15 and S15H samples.

### 3.5. CO_2_ Sorption Experiments at Different Temperatures

Prior to CO_2_ sorption experiments, each sample was outgassed at 373 K under high vacuum for 6 h. The adsorption temperature range utilized for these experiments was chosen between 273 and 298 K in step intervals of 10 K. Finally, the adsorption capacity of the amine-functionalized SiO_2_ substrates was determined experimentally, in an automatic adsorption instrument (Quantachrome Autosorb 1LC, Quantachrome Instruments, Boynton Beach, FL, USA). Additional textural and thermal (isosteric heat of adsorption) properties were, calculated from the corresponding CO_2_ adsorption isotherms evaluated at the different temperatures. CH_4_ adsorption at 273 K, on some of the silica samples, was also carried out with the aim of visualizing the selectivity of the modified SBA-15 adsorbents towards CO_2_.

## 4. Characterization Techniques

- FTIR: This technique will be helpful for realizing the presence of silanol and siloxane species on the surface of the adsorbents. FTIR signals were obtained from a Perkin Elmer Paragon 1000 (FT-IR) instrument equipped with an Attenuated Total Reflectance (ATR) tool.
- TGA: This analysis was performed on a Perkin Elmer Diamond instrument on a N_2_ flow of 50 mL·min^−1^, subjected to at temperature ramp of 5 °C·min^−1^ along a temperature interval of 323–823 K.
- Raman: These spectra are useful for providing an overall view of the structural consolidation of the adsorbents prepared in this work; in addition to the structure of the substrate itself, in particular, of silica bonds as well as free or vicinal silanol groups that lie on the silica surface. Through this technique it could be possible, in principle, to realize the arrangement of adsorbate molecules throughout the adsorbent surface. The instrument employed was a Raman Microscope (ThermoFisher Scientific, Waltham, MA, USA) with a 636 nm laser delivering a maximum power of 10 mW.
- TEM: Images were obtained from a HRTEM Jeol 2100F microscope (JEOL Ltd., Tokyo, Japan) operating at an acceleration voltage of 200 keV. This instrument was employed to observe the structural arrangement of the samples and to realize if the preparation procedure had affected (or not) the shape and structure of the SiO_2_ substrates.
- XRD: It is not only useful for checking the ordered hexagonal arrangement of the nanotubes making the SBA-15 hexagonal arrangement but also for determining the pore width and the thickness of their pore walls. The XRD parameters of the network were measured from a Bruker D8 Advance instrument (Bruker AXS, Madison, WI, USA) employing a Cu-Kα radiation wavelength of 1.54 Å in the low region (0.6° to 5.0 ° in the 2θ scale).
- N_2_ Sorption: The textural properties of the silica adsorbents were determined from an ASAP 2020 automatic instrument (Micromeritics Instrument Corp., Norcross, GA, USA), employing N_2_ at its boiling point (76 K at Mexico City’s altitude). The materials were previously outgassed at 373 K for 12 h. under a turbomolecular vacuum of 10^−6^ mbar. The surface area was determined from either the BET or *t*-methods, while the pore size distribution was obtained through the Non-Localized Functional Theory (NLDFT) approach. The kernel employed for this calculation was that corresponding to the filling of cylindrical pores along the boundary N_2_ adsorption curve at 77 K.
- NH_3_ TPD: The total acidity (Brönsted + Lewis) was qualitatively realized from a Micromeritics TPD/TPR 2900 NH_3_ programmed desorption (TPD) device (Micromeritics Instrument Corp.) provided with a TCD detector. The experimental procedure consisted in outgassing the sample under a mixture of N_2_ and air flowing at individual rates of 60 cm^3^·min^−1^ before being mixed from room temperature up to 773 K; the system was then kept at this temperature for 15 min. This was followed by the concurrence of N_2_ and NH_3_ flows of 60 cm^3^·min^−1^ each, up to a final temperature of 1073 K. Subsequently, the system was left to cool down to 303 K for about one hour under the same NH_3_ and N_2_ flows. Subsequently, an additional cleaning of the surface at 303 K was performed with a He flow of 60 cm^3^·min^−1^ during one hour. The definitive TPD experiment was carried out under a N_2_ flow of 60 cm^3^·min^−1^ used as a carrier gas and following to a heating ramp of 10 °C·min^−1^ up to 1073 K; while registering the amount of NH_3_ desorbed at each temperature.
- Solid NMR: Solid State Nuclear Magnetic Resonance experiments were run on a Bruker Advance II300 spectrometer (Bruker BioSpin, The Woodlands, TX, USA), operating at 59.62 MHz for ^29^Si. The magic angle spinning technique was altogether employed with the NMR results as a reference of tetramethylsilane (TMS). This technique is very important in order to ascertain the number of bonds established between the surface silanol groups and the ethoxy groups of the APTES functionalizing species.
- CO_2_ and CH_4_ adsorption and selectivity: Finally, CO_2_ and CH_4_ adsorption studies at different temperatures were performed on a Quantachrome Autosorb-1LC instrument; for this task, the samples were previously outgassed at 373 K for 6 h. CO_2_ adsorption isotherms were obtained from 263 to 303 K in order to obtain the CO_2_ enthalpy of adsorption at pressures from 0.001 to 1 bar.

## 5. Results and Discussion

### 5.1. Middle-Infrared Spectroscopy Studies

**Figure 1:**
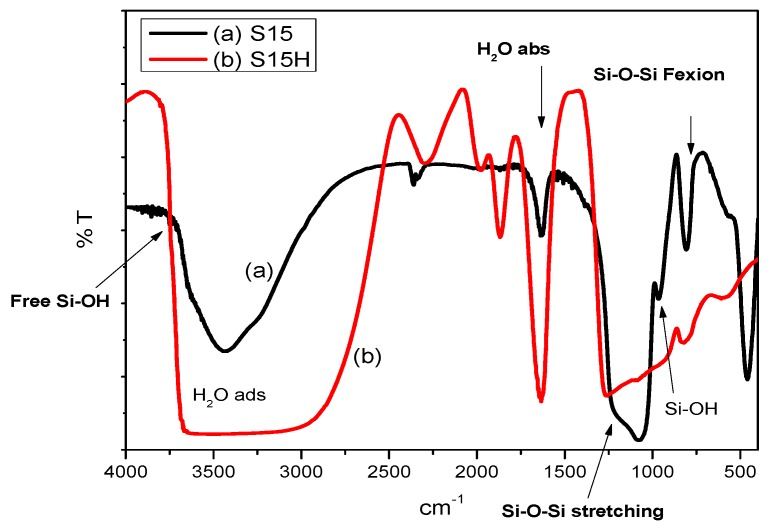
Fourier Transform Infrared (FTIR) spectra of: (**a**) Santa Barbara Amorphous (SBA)-15 precursor silica (S15); and (**b**) SBA-15 sample after being treated with 0.025 M NaOH at a temperature of 313 K (S15H).

The FTIR spectra in Figure 1 evidence vibrational changes of the molecules; therein, it is possible to note a prominent signal widening corresponding to physisorbed water in the S15H samples subjected to the hydroxylation treatment, if compared to the spectrum of the S15 precursor specimen. This difference surges even if all samples were previously annealed under vacuum at 473 K for 2 h. On the other hand, the intramolecular Si–OH (963 cm^−1^) and Si–O–Si flexion (802 cm^−1^) signals appear overlapped forming a broad band in the S15H spectrum. However, for hydroxylated samples, the band assigned to free silanol groups (Si–OH) appearing at 3769 cm^−1^ [32,33] results masked by the widening of the vibrational band of physisorbed water. Nevertheless, a larger amount of free SiOH groups sites interact with water through hydrogen bonds and promotes a rapidly hydration, which can be reflected in a larger amount of physisorbed water, again with respect to the same quantity in the S15 precursor sample.

**Figure 2:**
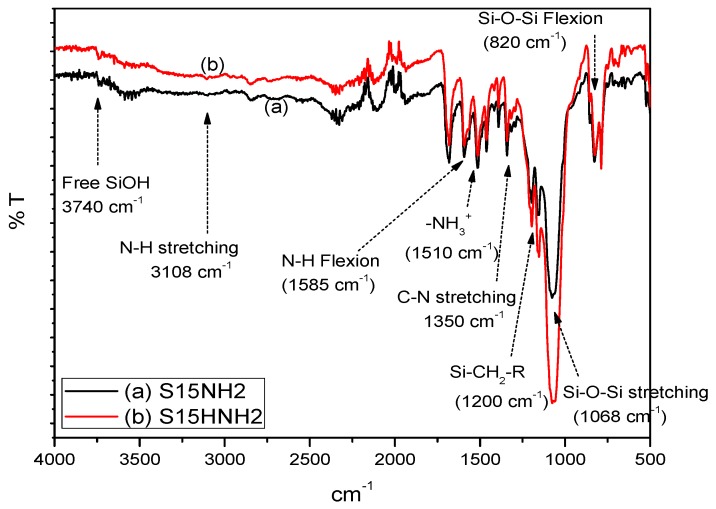
FTIR spectra of SBA-15 materials: (**a**) S15 precursor; and (**b**) S15H SBA-15 + NaOH 0.025 M samples, both characterized after functionalization with the same amount of 3-aminopropyltriethoxysilane (APTES).

The samples under scrutiny were now functionalized through a chemical bonding method, i.e., a process employing APTES and that was already described in the experimental section. In this case, a covalent bond is established between the surface of the substrate and the bridging amino species (i.e., APTES) (Figure 2). In this FTIR spectrum, it is possible to note silica vibrational bands as well as typical primary amine bands (–NH_2_), as in the case of APTES [34,35]. Notice that the bands corresponding to S15H, substrates previously subjected to a treatment with 0.025 M NaOH are the most intense of all, especially if these signals are compared to those obtained in the parent S15 specimen that is subjected to no alkaline exposure.

### 5.2. Raman Spectra of Precursor and Thermo Alkaline Treated Silica Samples

Raman spectra are useful in order to provide additional structural characterization, besides of that corresponding to the substrate itself, in particular to evidence the presence of free or vicinal silanol groups that are lying on the silica surface.

The traditional Raman spectra of vitreous SiO_2_ are characterized by bands appearing at around 410, 800, 1065, and 1200 cm^−1^ and are associated to vibrations of the amorphous silica network [36]. In our case, however, the bands that exist at around 410, 500, and 610 cm^−1^ can be linked to the vibrations of siloxane groups (Si–O–Si), which are assigned to rings of diverse sizes [37]. The signal at around 410 cm^−1^ is associated to the bending mode vibration of oxygen atoms inside a ring made of more than four silicon atoms (*n* > 4); this peak is usually known as the R band. The signal at around 490 cm^−1^ is associated to the breathing mode vibration of rings depicting four silicon atoms; this signal usually being known as the D_1_ band; the peak appearing at around 605 cm^−1^ is associated to the breathing mode vibration of rings formed by three silicon atoms, and branded as D_2_ bands [38]. Additionally, the peak at around 980 cm^−1^ is associated with the vibrational mode of (OH)-groups with respect to Si.

**Figure 3:**
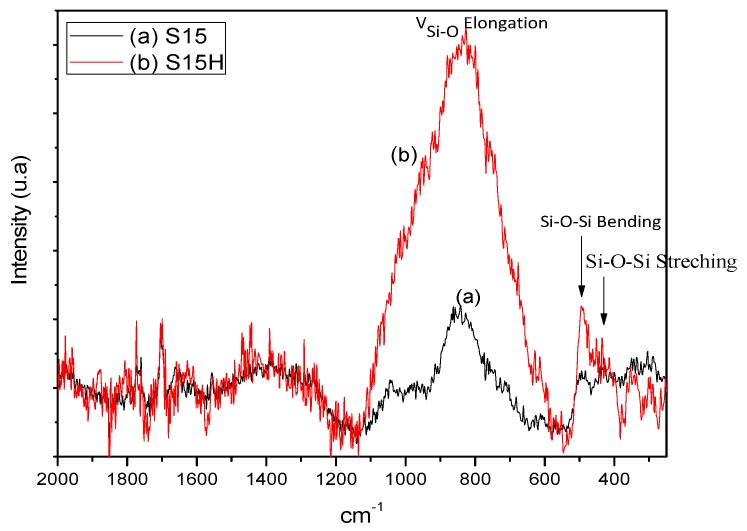
Raman spectra of SBA-15 materials: (**a**) precursor SBA-15 substrate (S15); and (**b**) silica after being treated with NaOH 0.025 M (S15H).

In the Raman spectra of the pristine S15 sample, it can be observed shallow R, D_1_, and D_2_ bands which are associated to rings of diverse sizes existing in the silica network (Figure 3). Additionally, the two bands appearing at around 850 and 1060 cm^−1^ can be linked [39] to the emissions located at around 850, 900, 950–1000, and 1050–1100 cm^−1^ and that can be due to tetrahedral silicon atoms having four, three, two or only one bridging oxygen atoms, respectively [40]. Non-bridging oxygen atoms could correspond to hydroxyl groups or to different metals, such as polysilicate alkaline cations [39]. In the Raman spectrum of the alkaline treated sample (S15H), an intense D_1_ band appears at around 490 cm^−1^. Besides, an intense and broad band appears from 600 to 1100 cm^−1^. This signal pathway suggests the existence of a slightly high population of silica rings formed by four silicon atoms. Furthermore, the intense band located at higher frequencies suggests the presence of a higher amount of tetrahedral silicon atoms having no bridging oxygens as consequence of the alkaline treatment to which the samples were subjected.

### 5.3. Thermogravimetric Analysis (TGA) Analysis of Precursor and Functionalized Silica Samples

**Figure 4:**
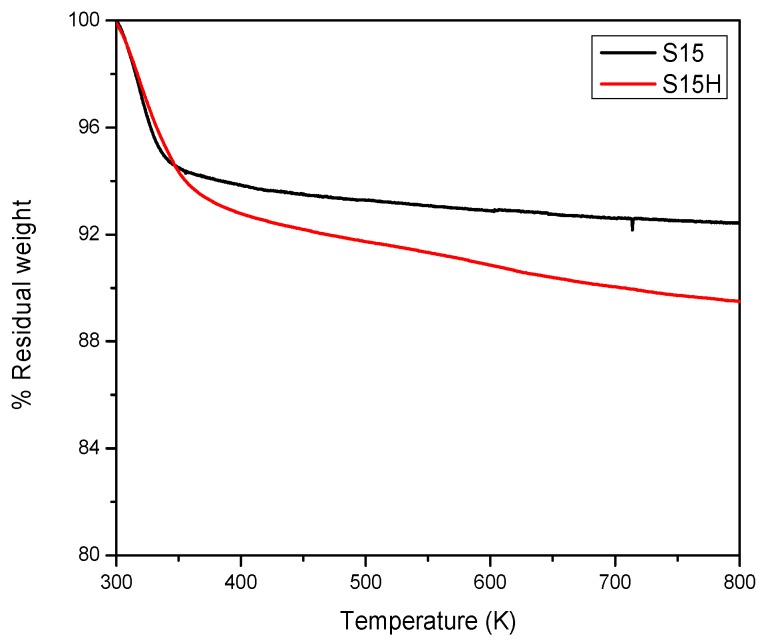
Thermogravimetric analysis under a N_2_ atmosphere of the S15 parent sample and of the S15H solid proceeding from the corresponding alkaline hydroxylation of the previous substrate.

The S15 precursor simple was subjected to TGA analysis (Figure 4). The weight loss of 6.0 wt % observed at around 373 K could be due to water desorption. Afterwards, a slightly decreasing slope is developed as the temperature is raised and in such a way that when the analysis is fulfilled at 823 K an additional mass loss of 2.0 wt % is detected. A slight mass loss of about 2.0 wt % is possibly due to the partial dehydroxylation of the SiO_2_ matrix, or in other words, the Si–OH groups condense to generate water molecules and Si–O–Si siloxane species, consequently the total mass loss is about 8.0 wt %. Nonetheless, for the S15H sample subjected to hydroxylation, a mass loss of 11.0 wt % arises thus confirming that this sample was more hydrated than the precursor solid. This, in principle, may be due to the fact that the hydroxylation treatment causes the rupture of siloxane groups to generate vicinal Si–OH groups, which can be easily rehydrated and, as the temperature is raised during the TGA analysis, become dehydrated promoting, at the same time, the condensation of them, since, as is well known, silica hydration-condensation processes are reversible phenomena [41].

**Figure 5:**
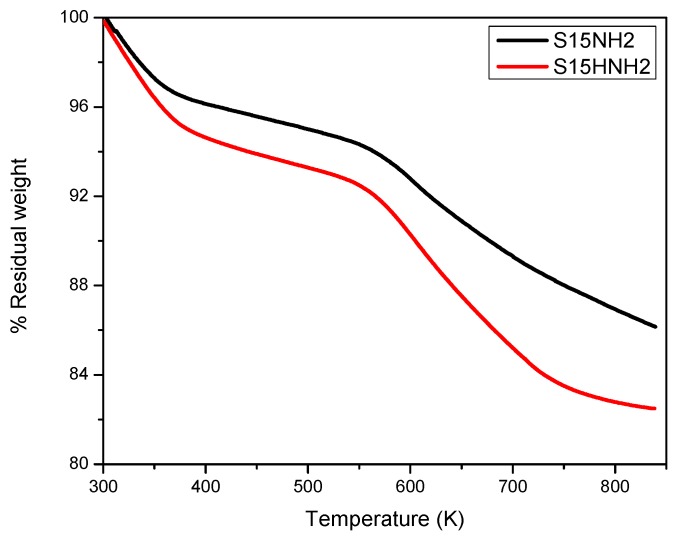
Thermogravimetric analysis, under N_2_ flow, of the S15NH_2_ and S15HNH_2_ APTES-functionalized silica specimens.

The TGA study corresponding to the samples functionalized with APTES are shown in Figure 5; namely, for the S15NH_2_ and S15HNH_2_ substrates. The S15HNH_2_ hydroxylated sample suffers the highest mass loss of all. The first TGA region is linked to desorption of the physisorbed water at around 373 K; i.e., 3.0 wt % for the S15NH_2_ specimen, and 5.0 wt % for the S15HNH_2_ solid. Nevertheless, the significant mass loss depicted from 543 K onwards, is caused by APTES thermal degradation. Specifically, the S15NH_2_ material presents a mass loss of 14.1 wt % while the S15HNH_2_ solid corresponds to a mass loss of 17.5 wt %. This implies a difference between these specimens of 19.4 wt %, which evidences that a major weight loss occurs for the hydroxylated and subsequently APTES functionalized simple.

### 5.4. X-ray Diffraction (XRD) Analysis at Low Angle

**Figure 6:**
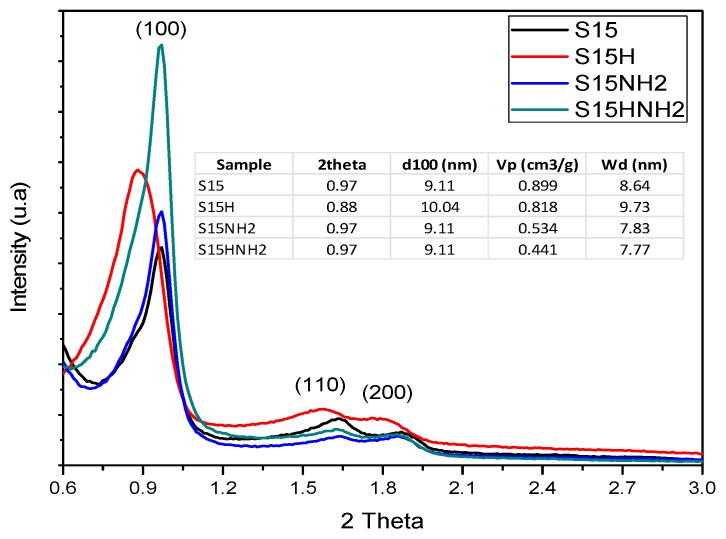
Low angle X-ray Diffraction (XRD) diffractograms of S15, S15H, S15NH_2_, and S15HNH_2_ solids.

The XRD diffractograms obtained for both precursor and functionalized silica materials are shown in Figure 6. Therein, it is observed for all cases, the structural parameters of a pore network consisting of cylindrical pore in a hexagonal packing with relatively sharp peaks appearing at (100), (110), and (200) planes, which are proof of this assertion.

As can be seen in Figure 6, there is no change in the interplanar 100 distances depicted by both S15 precursor and S15NH_2_ functionalized samples. However, when comparing the *W_d_* pore sizes obtained from Equation (1) [42] for the S15 and S15NH_2_ specimens, it is readily realized an evident pore size diminution provoked by the APTES presence inside the pores of the SBA-15 substrate.

(1) $$w_{d}=1.213\cdot d_{100}\cdot {\left(\frac{v_{p}}{\frac{1}{\delta }+V_{p}+V_{mic}}\right)}^{1/2}$$

where *V_p_* and *V_mic_* are the mesopore and micropore volumes, respectively, *δ* is the silica substrate density, and *d*_100_ is the 100 interplanar distance obtained from the XRD pattern.

In the case of the samples treated with alkaline solution, it can be noted a slight increase in the interplanar distance; this effect confirms that the hydroxylation treatment increases the pores size of the precursor solid without significantly altering the array and shape of cylindrical pores that constitute void volume of the substrate. Contrastingly, those samples functionalized with APTES suffered partial structural disarray, thus concluding that the S15HNH_2_ sample accept a highest amount of chemisorbed APTES in comparison to the S15NH_2_ sample, even if the two samples are put in contact with the same 3SiO_2_:2APTES molar ratio. This suggests that the hydroxylation process favored a large generation of free Si–OH sites. It is thus adequate to stress that the abundance and distribution of OH groups on the surface of silica adsorbents determine the occurrence of subsequent surface reactions.

### 5.5. Transmission Electron Microscopy (TEM)

**Figure 7:**
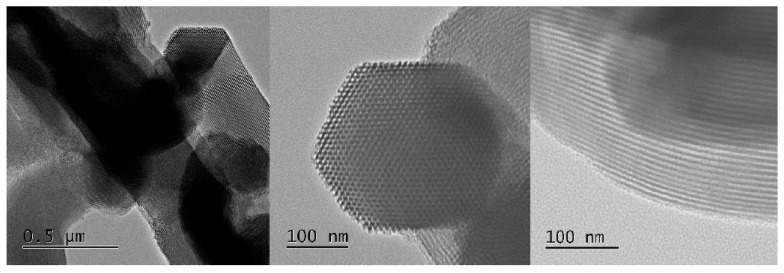
Transmission electron microscopy (TEM) images of the S15 precursor silica specimen.

The TEM micrographs shown in Figure 7 corroborate that the S15 precursor specimen consists of an ordered array of cylindrical capillaries disposed in a hexagonal packing, something that is evident in these images.

**Figure 8:**
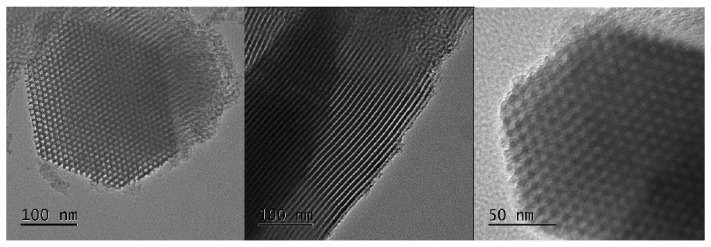
TEM images of the SBA-15 original sample, previously to the NaOH mild hydroxylation treatment, of the S15H hydroxylated specimen.

On the other hand, when the hydroxylation treatment is performed (S15H), there exist no obvious changes of the ordered silica pore arrangement with respect to the precursor S15 solid (Figure 8). This indicates, in a way similar to that suggested by the X-ray Diffraction analysis, that the sample subjected to the mild hydroxylation process, previously described, preserves the arrangement of its cylindrical voids and that only a slight lixiviation phenomenon occurs in a relatively uniform and soft manner. The TEM micrographs of the S15NH_2_ and S15HNH_2_ samples were not included in this manuscript because there did not exist obvious changes of the ordered silica pore arrangement with respect to the precursor S15 solid and the hydroxylated S15H samples (Figure 7 and Figure 8). Furthermore, it is important to remember that the APTES deposition was performed at low coverages.

### 5.6. N_2_ Adsorption at 76 K

**Figure 9:**
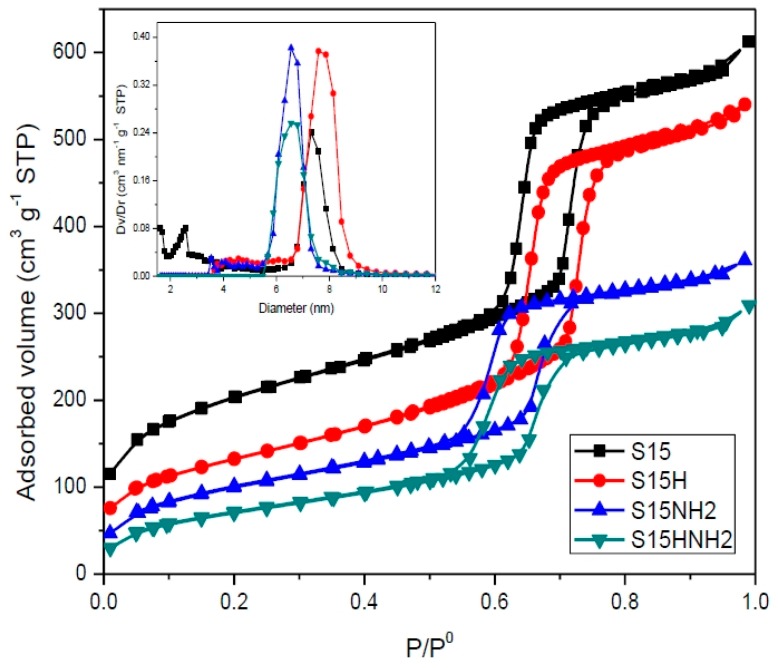
N_2_ sorption isotherms at 76 K and pore size distribution curves (figure inset) of SBA-15 substrates: S15, S15H, S15NH_2_, and S15HNH_2_. P/P^0^ is the relative pressure, while P^0^ represents the saturation pressure, finally STP means Standard Temperature and Pressure.

In Figure 9, N_2_ sorption isotherms at 76 K on precursor, S15, and hydroxylated, S15H, specimens and on their respective APTES functionalized solids (samples S15NH_2_ and S15HNH_2_) are displayed. The isotherms correspond to an IUPAC Type IV shape with an associated H1 hysteresis loop [43,44]. The figure inset in Figure 9, shows the pore-size distributions (PSD) of the same samples obtained by means of the Non Local Density Functional Theory (NLDFT) treatment for N_2_ desorption taking place along the boundary descending curve and according to a cylindrical pore model. Even if the dihydroxylation process is somewhat intense, because of lixiviation, the concentration of the caustic compound that was used to accomplish this end was sufficiently low as to keep the pore structure mostly unaffected, with practically no significant deformation of the pore shape. This can be also assessed by the close appearance of the XRD peak analysis. It is also pertinent to mention that the alkaline treatment, in the case of the S15 substrate, caused a pore width increment of 0.29 nm (see Table 1).

The main textural parameters of the different substrates were also calculated through the same N_2_ sorption analysis. The changes of the structural properties underwent by the S15 precursor sample can be due to both hydroxylation with NaOH and subsequent functionalization with APTES; these parameters are listed in Table 1. The temperature of 76 K corresponds to the approximate boiling point of N_2_ at Mexico City’s altitude conditions.

The above results confirm the beneficial effect of subjecting the surface of the substrates to a previous hydroxylation treatment that produces the elimination of micropores (i.e., the loss of *A*_mic_), and concomitantly, an increase of the mean pore size favored by the deposition of APTES on the surface of mesopores, while involving no pore mouth blocking. This happens since when SiO_2_ substrates are exposed to a hydroxylation process, some silica lixiviation occurs, the intensity of which depends on the concentration of the caustic solution; this process being known as alkaline desilication. It is also important to remember that the hydroxylation operation was performed at 313 K.

Now, when comparing the textural parameters listed in Table 1 and associated to the pioneering materials and their corresponding solids obtained after APTES functionalization of the surface, the next important effects are as follows:

(1) Compared to the precursor unhydroxylated substrate (S15), the presence of APTES has caused a decrease of the mean pore size in the S15HNH_2_ sample by 10.1%. Likewise, a decrease of 40.6% of the total pore volume. In turn, the S15H adsorbent depicted a pore size diminution of 13.2% and a reduction of 44.1% in the total pore volume. These facts suggest that more APTES molecules have been anchored on the hydroxylated specimen with respect to the unhydroxylated specimen.
(2) Additionally, the surface area decreased by 10.4% with respect to its original magnitude (S15). As the textural analysis shows, the S15H material depicts no micropores, thus suggesting that practically all the APTES molecules reside in the interior of mesopores and that they are uniformly dispersed on the surface. However, the disappearance of micropores from the S15 solid indicates that the access to these voids is somewhat precluded by the APTES species, perhaps anchored at pore entrances.

From the above statements, it is evident that the S15H adsorbent becomes the most efficient toward APTES capture if compared to the S15 parent specimen. The origin of this phenomenon may reside on the generation of higher number of free silanol groups (Si–OH) on the silica surface by action of the NaOH alkaline treatment.

### 5.7. NH_3_ Thermal Programmed Desorption from Silica Specimens (TPD-NH_3_)

**Figure 10:**
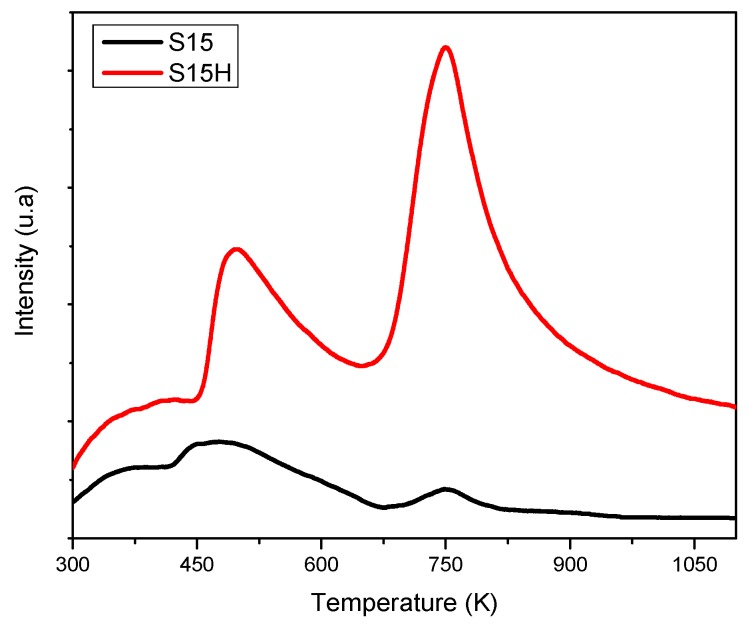
NH_3_ thermal programmed desorption from silica specimens (TPD-NH_3_) analysis of the S15 precursor solid as well as for the S15H hydroxylated material following thermo-alkaline treatment.

The amount of acid sites on the surface of the different adsorbents was determined by the amount of NH_3_ desorbed from the surface in the temperature range of 298–923 K. The results are schematically shown in Figure 10. In this figure, the silica solids depict two types of sites: (i) weak sites are evident by the band appearing from 443 to 673 K; and (ii) sites considered strong appear between 743 and 923 K. This implies the presence of Si–OH centers on the surface of the silica adsorbents, which are responsible of NH_3_ adsorption. The tendency found with respect to the abundance of acid centers, in our samples, is as follows: S15H >> S15. This result confirms that the hydroxylation treatment employed induces the formation of a greater number of acid sites, a fact that is reflected by the area displayed under each of the TPD peaks.

### 5.8. Nuclear Magnetic Resonance (NMR) Studies

**Figure 11:**
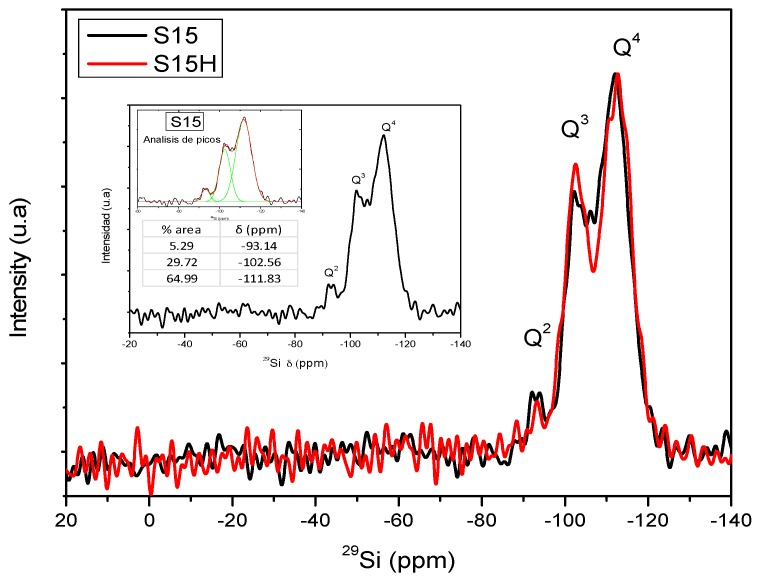
^29^Si Nuclear Magnetic Resonance (NMR) spectra (High Power Decoupling) for the S15 and S15H samples. The figure inset exemplifies a peak analysis performed after deconvolution of the set of different signals obtained through ^29^Si NMR analysis on the S15 solid.

**Figure 12:**
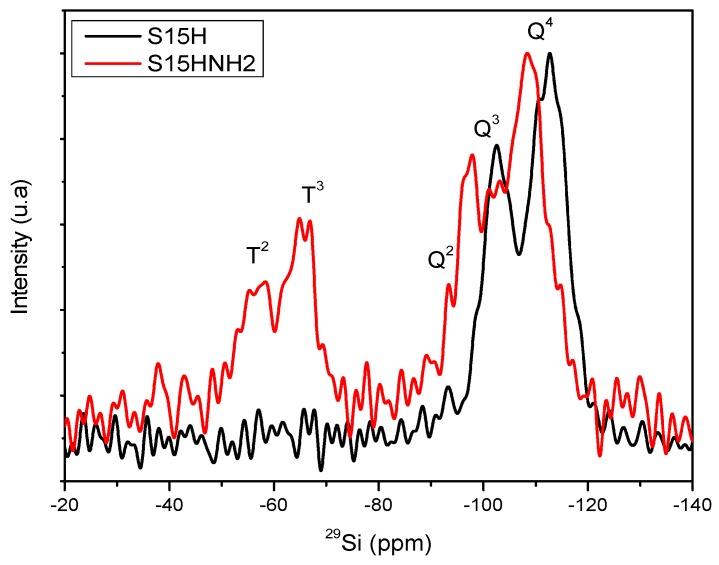
^29^Si NMR spectra (HPDEC) for the S15H precursor and APTES (S15HNH_2_) modified samples.

**Figure 13:**
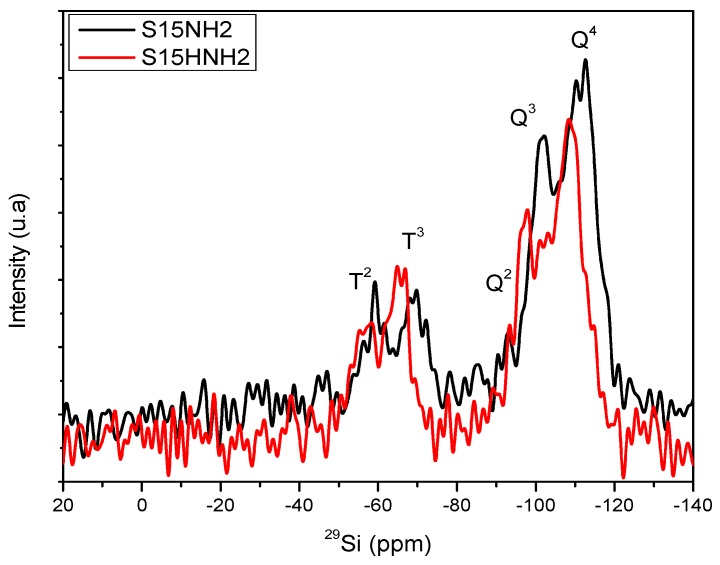
^29^Si NMR (HPDEC) spectra of solids modified on the surface via APTES: S15NH_2_ and S15HNH_2_ substrates.

Figure 11 shows an assessment of the High Power Decoupling (HPDEC) NMR spectra corresponding to the precursor, still unfunctionalized SBA-15 silica materials. On the other hand, Figure 12 depicts a comparison between the NMR spectra of the S15H hydroxylated substrate before and after being chemically functionalized with APTES. Finally, the NMR spectra displayed in Figure 13 shows a comparison of the signals emitted by the S15 (unhydroxylated) and S15H (hydroxylated) solids after being modified on their surface by the anchoring of APTES. All these NMR spectra exhibit signals appearing at chemical displacements of −112, −103, and −93 ppm and are due to the silica structure, and denoted as Q^4^, Q^3^, and Q^2^, respectively. The Q^2^, Q^3^, and Q^4^ bands are referred to silicon atoms bonded to two, three or four siloxane chains (–O–Si–) and the respective silanol groups (Si–OH) in order to complete four unions, respectively [45,46].

The ^29^Si NMR spectra (HPDEC) of the S15 and S15H samples, Figure 11 exposes similar population of Q^4^ and Q^2^ type silicon sites and an increased one of the Q^3^ type for the hydroxylated sample. This situation suggests that the pristine SBA-15 network maintains most of its silicon atoms totally integrated (condensed) into the network (Q^4^) while still possessing silicon atoms having one or two hydroxyl groups, but in lower amounts (Q^3^ and Q^2^); Furthermore, the above evidence suggests that hydroxylation promotes the formation of silicon atoms depicting a solitary hydroxyl group (Q^3^), as reveals the spectrum of sample S15H of Figure 11.

The signals centered at around −69 and −59 ppm observed in the spectra of Figure 12 and Figure 13 are associated to silicon atoms bonded to organic chains (Si–C) and labeled as T^3^ and T^2^ signals, which are equally distributed [45,46] and merge as consequence of the chemical bond established between the free silanol groups and the APTES functionalizing molecule. These bands can be attributed to the existence of silicon atoms bonded to carbon atoms and either two (T^2^) or three (T^3^) siloxane chains (Si–O–Si); additional surface hydroxyl groups that are required for completing the four unions of each silicon atom [45,46]. Therefore, therein it is evident the presence of T^3^ and T^2^ signals for the S15HNH_2_ solid, thus confirming the efficient anchoring of APTES on the surface of the precursor S15H material.

However, in the case of the ^29^Si NMR spectra in Figure 12 there can be also observed a slight chemical shift of the Q^4^ and Q^3^ bands for the case of the APTES functionalized sample (S15HNH_2_), with respect to the signals of the S15H hydroxylated solid (S15H). This could be due to a kind of screening effect because of the presence of APTES in the functionalized material, something that modifies the chemical ambient around the silicon atoms causing some shrinkage of the starting silica matrix. The emergence of a signal between the Q^2^ and Q^3^ bands in the spectrum of the S15HNH_2_ can be interpreted as consequence of the surface attaching of APTES, preferably trough one or two unions established over the silicon atoms having one hydroxyl group (Q^3^). This assumption is confirmed by the apparition the T^2^ and T^3^ groups. In other words, the attachment occurs by the substitution of one or two of the ethoxy groups (C_2_H_5_O–) of the APTES molecule with similar number of surface hydroxyl group (Si–OH) of the mesoporous substrate. Then, the slight increase of the Q^2^ population can be due to the integration of new silicon atoms of the APTES molecule with two remnant hydroxyl groups as consequence of the hydrolysis of their ethoxy groups. In order to avoid confusion, we have to remember that it is a well-known fact that the alkoxide groups of APTES or of any silicon alkoxide are readily substituted by the hydroxyl groups arising from water. However, in the absence of this substance, the only way for reaching condensation consists in the substitution of alkoxide groups by oxygen atoms proceeding from hydroxyl groups with the subsequent formation of the respective alcohol.

Figure 13 also depicts the same shifting effect, i.e., for the S15HNH_2_ substrate a chemical shift of about 4 ppm is evident with respect to the signals emitted by the S15NH_2_ solid [45,46]. Therefore, the S15HNH_2_ silica matrix provides emissions toward higher chemical displacements; this can be due to the change in the chemical environment around the silicon atoms or a contraction of the porous matrix caused by the APTES species existing on the pore walls of the previously hydroxylated specimen; this can be confirmed after N_2_ adsorption characterization.

This event is not surprising given that chemical displacements are very sensible to the chemical environment, and these conditions can be different even for the same molecule on the same support, depending on the amount of these chemisorbed species. In the same fashion, it is also evident in Figure 13 that the S15HNH_2_ solid possesses a slightly higher amount of T^3^ groups, if compared to those present in the S15NH_2_ specimen. This effect is also reflected on the relative decrease of the amount of available Q^3^ groups and due to Si–OH groups that have been employed to bind the APTES molecules to the surface. However, in this S15NH_2_ sample the T^2^ band displays a higher intensity than the T^3^ signal.

The intensity differences found between the S15NH_2_ and S15HNH_2_ samples confirm that a condensation reaction occurred between the hydroxyl groups (Q^2^ and Q^3^) on the respective surface of the substrate (S15 or S15H) and two or one ethoxy groups of APTES. For this reason, on the S15NH_2_ sample the most intense T^2^ band (having one hydroxyl group) and the increased Q^3^ band can only be associated to the presence of the silicon atoms of APTES. Additionally, the higher intensity of the T^3^ band in the S15HNH_2_ sample suggests a higher integration or condensation of the silicon atom of APTES as consequence of higher amount of vicinal silanol groups over the surface of the S15H substrate used.

Finally, in Table 2, the areal percentages associated to the Q and T NMR peaks are listed, once an appropriate deconvolution of these signal patterns has been performed (just as it was exemplified in Figure 11 for the case of the S15 sample).

The results listed in Table 2 confirm the statements induced from the aspects of Figure 11, Figure 12 and Figure 13. In this way, the argument advanced from the NMR characteristics of the S15 and S15H materials corroborates that these materials are appropriate for the anchoring of different amounts of APTES on their surface. Even if every synthesis is carried out, under the same conditions and employing the same amount of APTES, the S15H specimen adsorbs a larger amount of this compound. The net effect of hydrolyzing (S15H) is reflected in a larger proportion of Q^3^ species, i.e., free or adjacent silanol groups. There exists a difference of 15.6% in the amount of Q^3^ species between the S15 and S15H substrates. Besides, it can also be observed that the hydroxylated matrix appears to be less condensed than the matrix that was not subjected to this treatment; in this respect, just check the Q^4^ ratios against each other for the two materials.

In the same way, when comparing the functionalized solids, the largest amounts of T^2^ and T^3^ signals were found in the S15HNH_2_ sample rather than in the S15NH_2_ solid, something that can only be associated to the attached APTES species. Once the respective areal ratios are observed and considering the molar ratios chosen for carrying out the chemical anchoring on silica, as described in the Section 3.4, it is found (in a semiquantitative way) that 1.85 and 3.3 mmol of APTES have been anchored on the surface of the S15NH_2_ and S15HNH_2_ samples, respectively. This difference strongly confirms that the soft alkaline hydroxylation improves as much as 43.8% the anchoring efficiency of APTES towards the SiO_2_ surface (Table 2).

In brief, the hydroxylation alkaline process generates a higher efficiency toward the anchoring of APTES molecules than the unhydroxylated pioneering specimen does. This is mostly due to the larger amount of silanol groups on the pore walls of the hydroxylated samples. Under the presence of these species, the anchoring of the functionalizing amine species occurs more efficiently under molar ratios of 2:1 and 1:1, In contrast the unfunctionalized S15 precursor specimen is only able to anchor APTES molecules under a 2:1 Si–OH: APTES molar ratio.

### 5.9. CO_2_ Adsorption Studies and Determination of the Isosteric Enthalpy of Adsorption

**Figure 14:**
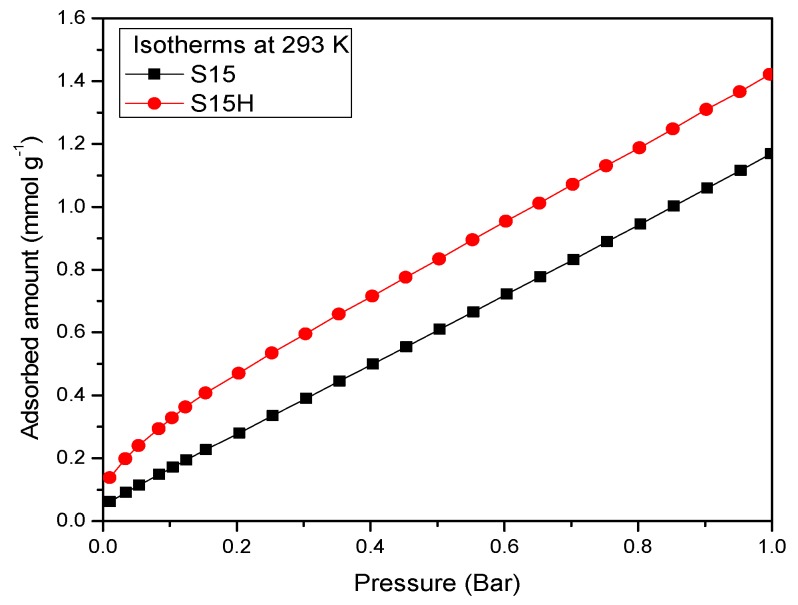
CO_2_ adsorption at 293 K, on SBA-15 precursor (S15) samples and hydroxylated (S15H) samples.

**Figure 15:**
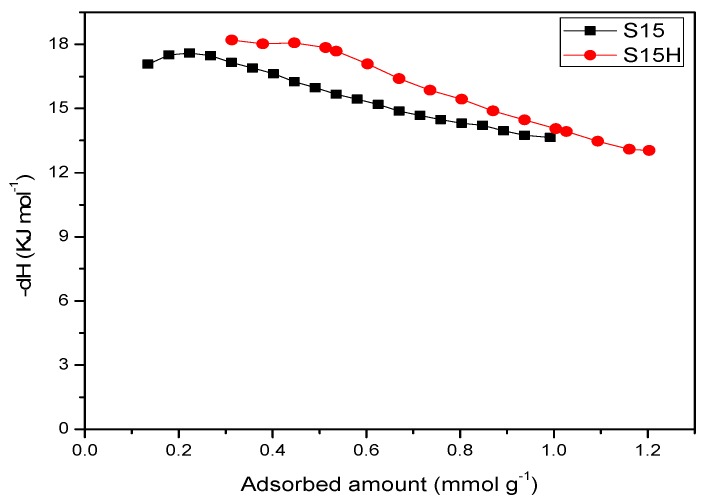
CO_2_ adsorption enthalpies of SBA-15 precursor and hydroxylated silicas (S15 and S15H).

**Figure 16:**
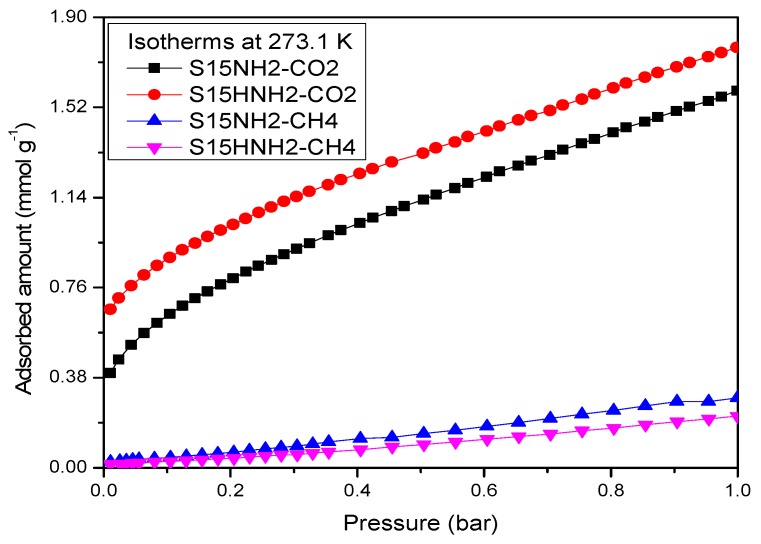
CO_2_ and CH_4_ adsorption isotherms at 273 K on APTES functionalized materials SBA-15 (pristine S15) and hydroxylated silica S15H.

CO_2_ adsorption isotherms on the assorted four silica substrates were determined at 263, 273, 283, 293 and 298 K or 303 K for each one of them (Figure 14, Figure 15 and Figure 16). When the isotherms of the unfunctionalized substrates (i.e., S15 and S15H) were analyzed, it was observed that the hydroxylation treatment endowed these adsorbents with a higher CO_2_ adsorption capacity (Figure 14). Nevertheless, the values of the isosteric enthalpy of adsorption evidence a relatively weak interaction (since these data are rather lower than the enthalpy of sublimation of CO_2_) between the adsorbent surface and the CO_2_ molecules. The possible reason for this behavior can be due to the intrinsic acidic nature of the silica surface [47]; there is no a drastic difference in the isosteric enthalpies depicted by the S15 and S15H silica samples Figure 15.

**Figure 17:**
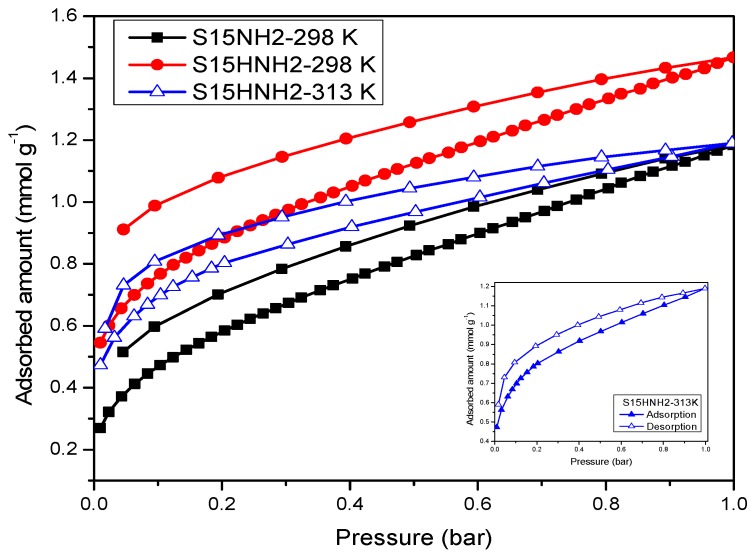
CO_2_ adsorption isotherms at 298 K on APTES functionalized materials SBA-15 (pristine and previously treated SBA-15) and isotherm at 313 K for the sample SBA-15 hydroxylated and functionalized with APTES. In all cases the adsorption and desorption processes depict upper and lower pathways, respectively.

On the other hand, the CO_2_ adsorption isotherms corresponding to the APTES functionalized substrates confirm that the hydroxylated specimens better contribute to a more efficient and energetic CO_2_ capture at different temperatures (Figure 16 and Figure 17). In addition, the affinity toward CO_2_ of amino-functionalized silica substrates (hydroxylated or not) is better than that depicted by the unfunctionalized solids.

Figure 16 displays a comparison between CO_2_ and CH_4_ adsorption at 273 K on the precursor and hydroxylated amino-functionalized silica materials. Therein, it can be appreciated that the aforementioned substrates present a strong selectivity toward CO_2_ rather than to CH_4_. This could be due to the fact that the methane molecule is non-polar and it is not establishing a meaningful attractive interaction with the amine-functionalized SiO_2_ surface. The adsorbed volumes shown by the silica samples toward CH_4_ are too low for being reflected in a significant CH_4_ uptake. These contrasting adsorption characteristics between CO_2_ and CH_4_ will indeed be very useful (i.e., selective) for achieving a fine separation of mixtures including these gases. Likewise, note the better affinity at 298 K towards CO_2_ depicted by S15HNH_2_ results to be 19.2% higher than the amount adsorbed by the S15NH_2_ matrix at 1 bar. However, even at 298 K and 0.15 bar, there is a difference between the two samples of 36.2% (Figure 17). Even at 0.15 bar, S15HNH_2_ shows a better performance at 313 K than the S15NH_2_ specimen, thus stressing this way that the thermos-alkaline treatment, previous to APTES surface functionalization, is very convenient in order to significantly raise CO_2_ capture capacity with respect to the amount adsorbed by the APTES functionalized but not previously hydroxylated S15NH_2_ substrate. Therefore, the S15HNH_2_ solid is more recommendable than the conventional (i.e., not hydroxylated) samples for pursuing enhanced CO_2_ adsorption this effect being valid at 0.15 bar and temperature range of 318–348 K [48]. It is also important to mention that the CO_2_ isotherms shown in Figure 17 depict low-pressure hysteresis loops at 298 K. However, this phenomenon is more accentuated for the S15HNH_2_ specimen than for the S15NH_2_ material. This suggests that there may exist a stronger interaction between CO_2_ and the hydroxylated surface (before being functionalized with APTES) than with the analogous but unfunctionalized sample [49].

Importantly, the S15HNH_2_ even if substrate presents the best performance in terms of CO_2_ adsorption. The total adsorbed volume of this gas is not too high when compared to other SBA-15 materials reported in the literature. Nevertheless, this comparison become relative ever since the overall CO_2_ capture capacity depends on a number of factors, among them:

(1) The synthesis conditions and the method employed to eliminate the templating block copolymer to leave behind the pore entities. This process is intrinsically linked to the amount of SiOH groups on the surface [50,51].
(2) The method of amine deposition on the silica surface (in-situ or ex-situ) [52,53].
(3) The nature of the amine molecule to be surface deposited on the pore walls [54,55].
(4) The quantity of amine compound employed for the surface functionalization [35].
(5) The functionalization method (e.g., impregnation, chemical anchorage or a combination of both). Olea et al. [56] have taken into account most of the above factors, including the exploration of different surface functionalization approaches of SBA-15 materials.
(6) The experimental conditions selected for CO_2_ capture (e.g., temperature, pressure, CO_2_ flow rate, etc.).

Nonetheless, the present work demonstrates that the alkaline pretreatment is adequate for favorably allowing the polarity of the conventional SBA-15 surface in such a way that the substrates becomes more appropriate for being subjected to further reactions with amine species without compromising the thermal stability of the pore substrate at temperatures lower than the calcination one.

On the other hand, in the Section 3.4 the APTES quantity that was used for functionalizing of surface of the parent solids. i.e., S15 and S15H is relatively small (3SiO_2_:2APTES, e.g., for preparing 150 mg of either S15 or S15H sit is required a volume of 0.4 mL of APTES). This action is useful with the aim of elucidating the way by which the APTES molecules interact with the surface at low surface coverage. However, eventually, analogous experiments could be performed by slightly increasing the APTES:SiO_2_ molar ratio as to increase the concentration of APTES molecules on the surface of silica substrates.

**Figure 18:**
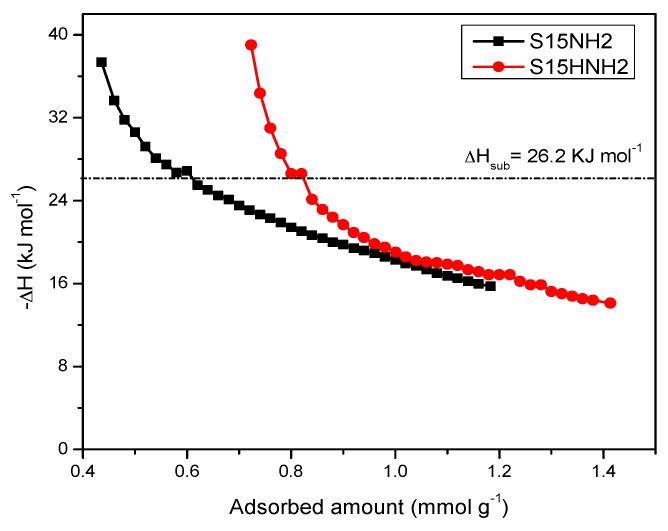
CO_2_ enthalpy of adsorption of the APTES functionalized SBA-15 substrates ((pristine and previously treated SBA-15).

Finally, in order to know the forces that dominate the adsorbate-adsorbent interactions, a study of the isosteric enthalpy of adsorption was performed in relation to CO_2_ adsorption at different temperatures; this study was carried out for the solids modified on the surface with APTES. The adsorption runs were made at the following temperatures: 263, 273, 288 and 298 K; the determination of the isosteric enthalpy of adsorption was calculated from a Clausius–Clapeyron type equation (which simulated the quasi-solidification of CO_2_ on the silica surface) (Figure 18). The results shown with respect to the enthalpy of adsorption of CO_2_, for the APTES functionalized substrates, emphasize that in these materials prevails an adsorption potential that strengthen the adsorbate-adsorbent forces, in comparison to the process carried out on the unfunctionalized silica solid. Then, the thermo-alkaline pretreatment and its subsequent functionalization with APTES lead to a higher capacity for CO_2_ adsorption in comparison to that achieved by the amine-functionalized silica precursor specimen. The absolute −*ΔH* values result higher than the CO_2_ enthalpy of sublimation (*ΔH*_sub_) at 298 K and 1 bar (i.e., 26.2 kJ·mol^−1^). These results constitute an evidence of that a larger amount of CO_2_ is being anchored on the surface of S15HNH_2_ solid than on that of the S15NH_2_ adsorbent. Even more, the S15HNH_2_ adsorbent establishes a stronger interaction with CO_2_ than the unhydroxylated specimen, which is extended up to large surface coverages. For instance, for a CO_2_ surface coverage of 0.72 mmol·g^−1^, an enthalpy of adsorption of −23.2 kJ·mol^−1^ is found for the S15NH_2_ specimen in contrast to the −39.2 kJ·mol^−1^ evolved by the S15HNH_2_ adsorbent.

In addition, the chemical reason for this adsorption behavior can reside on the evidence provided by the NMR spectra; the ideal case would be that three Si–OH silanol groups be required to substitute the three alkoxi groups of the APTES molecule. However, this occurs in such a way that the remaining Si–OH species still be available for establishing more bondings with more alkoxi groups proceeding from other APTES molecules. That is, the attaching of one APTES molecule render new Si–OH groups for the attachment of other APTES molecules. The above behavior can be attributed to the chemical nature of the adsorbent–adsorbate pair. There are two possible chemical explanations for these phenomena to occur:

(a) The basic property of amino functionalized silica acts as a Lewis base that provides an electron pair to the CO_2_ molecule, i.e., a coordination link is formed. The adsorbed CO_2_ molecule then establishes additional coordination links with the hydrogen or oxygen atoms proceeding from adjacent APTES or with the hydroxyl groups of the silica substrate.
(b) When the surface population of Si–OH species is increased via the hydroxylation treatment, the APTES molecules exist over larger domains of the pore structure, therefore a larger amount of CO_2_ molecules gets captured. This last process could be carried out by the zwitterionic reaction that renders ammonium carbonate after the reaction of amine and CO_2_ species [57]. The capture of CO_2_ by neighboring (i.e., more concentrated) amine species results to be more stable than if only an individual, isolated amine species reacts with CO_2_. A proton of one of the neighboring amines is transferred to the adjacent one; the former NH_2_ group attracts the carbon dioxide to it due to its refurbished electron density. This is a reflection of the assertion, since therein a considerable enthalpy of adsorption is attained by the amine-functionalized and pre-hydroxylated SBA-15 species (Figure 18).

## 6. Conclusions

The present investigation shows that a soft alkaline hydroxylation is suitable for raising the population of Si–OH surface groups in conventional silica substrates; something that could be due to the breaking of Si–O–Si species into groups and, at the same time, involving the loss of micropores while preserving the mesoporous structure. For example, in the case of the S15 substrate, the alkaline treatment caused a pore width increment of 2.9 Å, which resulted appropriate for our purposes. In any case, these additional Si–OH groups allow the attachment on the surface of diverse species, such as the APTES which can help to fix a myriad of active chemical species over mesoporous substrates.

The studies of the amount of acid sites on the surface of the different adsorbents determined by the TPD-NH_3_ desorption and ^29^Si NMR confirm that the hydroxylation treatment induces the formation of a greater number of acid sites. In this way, the argument advanced from the NMR characteristics of the S15 and S15H suggests that these materials are appropriate to anchor different amounts of APTES on their surface, even if every synthesis is carried out under the same conditions and employing the same APTES: solid molar ratio. This is likely due to the abundant presence of Q^3^ species mainly, i.e., free or adjacent silanol groups on which the bonding of APTES occurs and that allows the functionalization of the surface with other important chemical species. The ^29^Si NMR analysis reveals, in a semiquantitative way, that 1.85 and 3.3 mmol of APTES have been anchored on the surface of the S15NH_2_ and S15HNH_2_ samples, respectively. Furthermore, the fixing of APTES over these substrates preferably occurs through only one chemical union. Distinctively, for the case of non-treated SBA-15 substrates, the APTES species remain fixed to the silica network through either one or two chemical bonds. These results demonstrate the effectiveness of the alkaline treatment for increasing the population of Si–OH anchoring sites without affecting the structure of mesoporous substrate.

Finally, the adsorption enthalpy of the APTES-functionalized substrates, confirms a higher CO_2_ adsorption when the silica substrates are previously treated with an alkaline solution. All the evidence shown herein suggests that the interaction of CO_2_ molecules with the systems treated with alkaline solutions could be due to the existence of a larger amine group population, arising from the APTES species. However, the adsorption of CO_2_ could be the result of combined interactions with the hydroxyl groups of remnant and vicinal S–OH groups existing on the substrate or coming from APTES. Although the amount of CO_2_ retained in the systems herein described could result to be similar or lower than those obtained in other investigations, the possibility of optimization is still attainable.

## Figures and Tables

**Table 1:** Textural properties obtained from N_2_ sorption at 76 K.

Sample	*A*_BET_ (m^2^·g^−1^)	*V*_t_ (cm^3^·g^−1^)	*A*_ext_ (m^2^·g^−1^)	*A*_mic_ (m^2^·g^−1^)	*D*_mode_ (nm)
S15	696.4	0.899	456.4	240.0	7.30
S15H	466.9	0.818	439.7	27.2	7.59
S15NH_2_	360.9	0.534	335.8	25.1	6.56
S15HNH_2_	260.9	0.441	260.9	0.0	6.56

*A*_BET_ is the Brunauer-Emmett-Tell (BET) surface area, *V*_t_ is the total adsorbed volume, *A*_ext_ is the external surface area, *A*_mic_ is the virtual micropore surface area and *D*_mode_ is the modal pore diameter arising from the Non Local Density Functional Theory (NLDFT) approach by employing a kernel that utilizes the boundary desorption isotherm relative to N_2_ at 76 K in cylindrical pores.

**Table 2:** Areal percent of signals proceeding from Nuclear Magnetic Resonance (NMR) spectra obtained from the deconvolution of the respective signals of precursor and amine-functionalized substrates.

Material	% Q^2^ Area	% Q^3^ Area	% Q^4^ Area	% T^2^ Area	% T^3^ Area
S15	5.29	29.72	64.99	-	-
S15H	2.87	35.28	61.85	-	-
S15NH_2_	0.16	32.84	48.75	7.91	10.34
S15HNH_2_	5.30	21.25	45.00	13.54	14.90
